# Aortic Valve Replacement in the Current Era

**DOI:** 10.3390/jcm14051447

**Published:** 2025-02-21

**Authors:** Shannon Parness, Jack T. Womble, Tori E. Hester, Panagiotis Tasoudis, Aurelie E. Merlo

**Affiliations:** Division of Cardiothoracic Surgery, Department of Surgery, School of Medicine, University of North Carolina at Chapel Hill, Chapel Hill, NC 27599, USA; shannon_parness@med.unc.edu (S.P.); jack_womble@med.unc.edu (J.T.W.); tori_hester@med.unc.edu (T.E.H.); tasoudis@ad.unc.edu (P.T.)

**Keywords:** aortic valve replacement, aortic valve disease, surgical aortic valve replacement, transcatheter aortic valve replacement

## Abstract

Aortic valve disease (AVD) is a highly prevalent condition worldwide. Aortic valve replacement (AVR) is the surgical treatment for those with severe disease. Common etiologies of AVD include aortic stenosis (AS), aortic insufficiency (AI), endocarditis, and congenital diseases. Shared decision-making plays a large role in the treatment methodology chosen for each patient. Selection of valve type and surgical intervention requires strong considerations of age and compatibility with vitamin K antagonists (VKAs) to ensure optimal post-operative outcomes. Due to the development of novel surgical techniques, including transcatheter AVR (TAVR) and placement of sutureless valves, patients who previously had limited access to AVD surgical options can now be considered for AVR. Further research into therapeutic development is imperative to improve patient short- and long-term outcomes as well as widen surgical candidacy for those seeking AVR for the management of AVD. Overall, AVR will continue to hold its prominent role in the treatment of AVD.

## 1. Brief Introduction to Aortic Valve Replacement

Aortic valve replacement (AVR) is a surgical treatment for people who have a severely diseased aortic valve (AV). The disease etiology can be aortic stenosis (AS), aortic insufficiency (AI), endocarditis, and congenital diseases such as a bicuspid aortic valve (BAV). AS is the most common valvular heart disease (VHD) in the developed world, accounting for two-thirds of VHD deaths between 1999–2020 [1]. Many studies have found that the prevalence of AS is age-dependent [1,2,3], and with an aging population in the United States, the prevalence is up to 4.6% in adults older than 75 years old [3]. AI is also associated with increasing age in developed countries but is found to be increasingly associated with infectious etiologies in developing countries [4,5]. In the Framingham study, the prevalence of AI was estimated to be up to 4.9% [5]. In today’s age, there are many options on how to surgically treat AV disease (AVD), along with many valve options that are available to use. Surgical options include surgical AVR (SAVR) and transcatheter AVR (TAVR). The most common valve types that are available include mechanical and bioprosthetic valves. Overall, AVR is an indispensable treatment option for those with AVD.

## 2. Indications for Aortic Valve Replacement

### 2.1. Overview

AVD has multiple etiologies, including AS, AI, and endocarditis. All three etiologies are best evaluated with a transthoracic echocardiography (TTE) [6]. While medical management can be used to treat these conditions temporarily, surgical treatment is considered a primary long-term treatment option, especially in severe diseases. Furthermore, there have been many advances in the surgical options for AVR. TAVR became a popular treatment option in 2012 [1], which was highly beneficial for those who were considered inoperable. The selection of the type of valve to use has also evolved as new technologies continue to emerge. The 2020 American College of Cardiology (ACC)/American Heart Association (AHA) guidelines, and the 2020 European Society of Cardiology (ESC)/European Association for Cardio-Thoracic Surgery (EACTS) guidelines, will be referenced throughout this paper, with specific classes and levels of recommendations listed in Table 1, Table 2, Table 3, Table 4, Table 5, Table 6, Table 7, Table 8 and Table 9.

### 2.2. Aortic Stenosis

AS is the most common VHD in the developed world, accounting for 62.1% and 61.7% of VHD deaths between 1999 and 2020 in females and males, respectively [1]. The progression of AS is variable between patients, with many being asymptomatic. However, studies have found that 75% of patients die within three years of symptom onset if no interventions are performed [7]. Due to the increasing disease severity with age, current guidelines state that AVR is indicated in patients that have severe symptomatic AS, and severe asymptomatic AS with a left ventricular ejection fraction (LVEF) of less than 50% [3,6]. It has been observed that there is a mortality benefit to performing AVR for those with mild AS who have LV dysfunction, especially as studies suggest myocardial fibrosis occurs with the progression of AS [7,8]. Samad et al. found that patients with moderate or severe AS with LV systolic dysfunction (LVSD) had a lower mortality rate when they received AVR compared to treatment with medical therapy alone [9]. Kang et al. evaluated the outcomes of early surgery with AVR vs. conservative care in asymptomatic patients with severe AS. They found that the early surgery group had a decreased incidence of death from cardiovascular causes as well as a decreased incidence of all-cause mortality compared to the conservative care group [2]. Sevilla et al. also reported on the timing of intervention for asymptomatic patients with AS. These authors state that with the improving outcomes and the decreasing rates of complications of surgical interventions, intervening before symptoms begin is reasonable [10]. Patients with AS, even when asymptomatic, can begin to lose functionality of the LV. This can lead to many deleterious consequences, and as such, eventual surgical repair is largely expected.

### 2.3. Aortic Insufficiency

AI is defined as the regurgitation of blood into the LV due to insufficient closing of the AV [4]. This disease has a prevalence of up to 4.9%, which increases with age, and accounts for greater than 50% of AVRs in the United States [5]. AI can be an acute or chronic manifestation. Acute manifestations are typically due to infective endocarditis (IE), aortic dissection, and traumatic rupture [4,5]. Chronic AI can be due to calcific disease, BAV, connective tissue diseases, and rheumatic heart disease [4,5]. Acute AI is a higher acuity situation as severe LV overload can occur very rapidly, leading to pulmonary edema, hypotension, and cardiogenic shock. Therefore, early surgical intervention of acute AI is necessary and should not be delayed [5,6]. Current guidelines state that patients with severe symptomatic AI should obtain an AVR regardless of LV status, and patients with severe asymptomatic AI should obtain an AVR when LVEF is less than 55% [6]. While acute AI requires surgical intervention immediately, chronic AI can be asymptomatic as LV remodeling occurs over time. This, however, can lead to heart failure, and surgical intervention may be warranted at this stage [5]. AVR is a highly important treatment option for those with AI, especially in acute situations when surgery is necessary.

### 2.4. Endocarditis

Endocarditis is broadly defined as an infection of the endocardium. IE is a rare condition; however, it carries a 15–30% mortality rate [11,12,13]. Prior conditions, such as rheumatic heart disease, degenerative valve disease, intravenous drug use, and congenital heart diseases, are all key risk factors for the development of IE [11]. In order to treat IE, broad antibiotic treatment against the pathogen needs to be initiated, and further analysis of surgical intervention should be evaluated by a Multidisciplinary Heart Valve Team (MDT) [6]. Other etiologies of endocarditis include nonbacterial thrombotic endocarditis, Libman-Sacks endocarditis, vasculitis, and connective tissue disease [12]. Rather than being caused by an infection, these conditions may be due to hypercoagulable states, systemic lupus erythematosus, and congenital disorders [12]. Surgical intervention is indicated when patients present with valvular dysfunction, complications such as conduction abnormalities, aortic abscesses, or destructive lesions ensue, and when patients have persistent or recurrent infections [6,12]. Up to 50% of patients will require surgical intervention due to heart failure, failure to control the infection, and failure to prevent septic emboli [13]. Endocarditis, particularly IE, is a disease with many complications and carries a poor prognosis. Antibiotic treatment and surgery are part of the limited options patients have to treat this disease.

## 3. Selection of Valve Type

### 3.1. Overview

**Figure 1:**
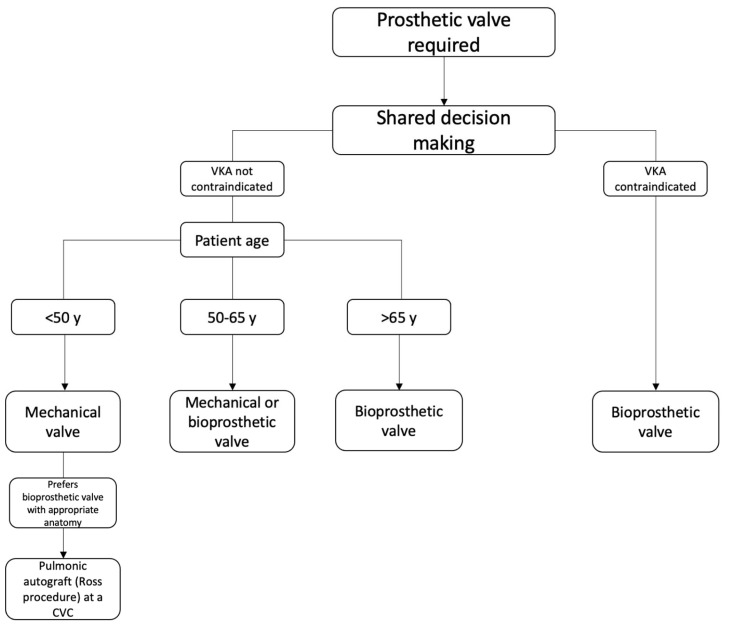
Adaptation of the 2020 ACC/AHA Guidelines for Valve Selection. Acronyms: ACC: American College of Cardiology, AHA: American Heart Association, VKA: Vitamin K Antagonist, CVC: Comprehensive Valve Center.

The selection of valve type in AVR depends on many factors. Above all else, shared decision-making is the largest consideration in this process [6]. This is because the selection of valve type comes with significant lifestyle changes for the patient. The main valve types that are available include mechanical, bioprosthetic, and pulmonic valve autograft—the Ross procedure. Sutureless valves are subtypes of bioprosthetic valves, and TAVR is a transcatheter delivery option for bioprosthetic valves. Other considerations in deciding which valve to use include age, life expectancy, and medication adherence [14]. These considerations are highly important in ensuring a successful valve replacement for these patients. A simplified decision process is outlined in Figure 1.

### 3.2. Mechanical

The first successful AVR was performed in the 1960s using a mechanical valve [14]. There have been many advances in the types of mechanical valves available since then. For example, the caged ball valve, which is a metal cage with a ball inside that changes positions based on pressure within the chamber, is no longer used due to the high risk of clotting and adverse hemodynamic effects. Current models include the tilting disk valve and the bileaflet valve. These are less thrombogenic, which is highly important in mechanical valves where thromboemboli are the main concern post-procedure [14,15]. Therefore, lifelong anticoagulation using vitamin K antagonists (VKAs) is a requirement for patients receiving this valve [6,14,15] and major bleeding events are the largest consequence [15]. Virchow’s triad describes the etiology of thrombus formation. Mechanical valves generate thrombus from endothelial damage introduced by the surgery and the creation of local turbulent flow that leads to stasis. Any thrombus formation on the valve has the ability to embolize [16]. As stated above, age is a highly important consideration when deciding if a patient should receive a mechanical valve or not. Mechanical valves are known for their longevity, lasting over 20 years [14]. Therefore, patients less than 50 years old should be considered for a mechanical valve as long as there are no contraindications to VKA therapy [6].

### 3.3. Bioprosthetic

Bioprosthetic valves are generally made of bovine pericardium or porcine aortic valves, but they can also be made of equine or porcine pericardium [15,17]. These valves can be stented, and reinforced with a stent frame and a sewing ring, or stentless, lacking the stent frame and sewing ring [17]. The main consideration in receiving a bioprosthetic valve is age. Structural valve degeneration (SVD) is a known consequence; this arises from calcification of the tissue leading to valve dysfunction, therefore decreasing its durability [17]. The 15-year risk for reoperation is 22%, 30%, and 50% for patients receiving the valve at 50, 40, and 20 years old, respectively [6]. Therefore, patients older than 65 years old, or those with a contraindication to VKA therapy, are recommended to receive a bioprosthetic valve [6]. More research into the pathophysiology of SVD is necessary to mitigate long-term risks for these patients, especially as the use of bioprosthetic valves is increasing compared to mechanical valves [17].

### 3.4. Sutureless Valves

Sutureless valves are types of bioprosthetic valves that do not require extensive sutures to anchor them into place (although they do still require placement of sutures) [18]. Three are commercially available for use: the 3F Enable, Perceval S, and Intuity Elite. The diseased valve is excised prior to the positioning of these valves. The 3F Enable requires one suture while the Perceval S requires none. These both work using a nitinol metal frame that is positioned and deployed into the valve for adherence [18]. The Intuity Elite valve is a balloon-expandable valve that is stainless steel and cloth-covered. It works by using balloon catheter guidance for the correct positioning; then it is expanded to the correct annular size and is secured with three sutures [18]. As these valves do not need extensive suturing, the operation time and cross-clamp time are decreased [18], making these valves great options for those who cannot undergo cardiopulmonary bypass (CPB) for an extended amount of time.

An important consideration prior to using sutureless valves is the size of the aortic annulus, and therefore the size of the valve. As the anchoring process is mainly achieved by correct positioning before deploying the valve rather than the use of sutures, paravalvular leak and valve migration are rare complications [18]. Studies have shown that the incidence of paravalvular leaks, about 2–4%, correlates with the learning curve required for this surgery and is significantly reduced over time [18]. Another possible consequence includes conduction disorders. To investigate this, van Boxtel et al. observed 31 patients who underwent sutureless AVR with Perceval S in the Netherlands. They found that 11 patients developed a new left bundle branch block (LBBB) and 4 patients required permanent pacemaker placement due to complete atrioventricular block [19]. Pollari et al. reported on their single-center experience of using the Perceval sutureless valve series with a 10-year follow-up. Their study population had a mean age of 76.4 years. The median survival was 7.76 years and the median timing of remaining SVD-free was 10.3 years. Importantly, this study notes that younger age is a risk factor for developing SVD [20]. The use of sutureless valves has become more popular with the increasing use of TAVR. This could possibly be due to an increasing number of patients, even those not classically considered candidates for AVR, being referred for TAVR, and if their anatomy is not amenable, sutureless valve placement can still be an option. However, it is still a relatively new surgical option, and therefore, more research is needed to investigate the risks and benefits of this procedure, both short-term and long-term, especially in varying age groups.

## 4. Types of Procedures

### 4.1. Transcatheter Aortic Valve Replacement

A transcatheter heart valve was first described in 1992 by HR Andersen et al. [21,22]. Currently, the two most common valves used in TAVR are the SAPIEN 3, a balloon-expandable valve, and the Evolut, a self-expandable valve [23]. The design of these two valves has contributed to the development of TAVR as a safe, routine procedure [24]. Four different sizes of the SAPIEN 3 valve are available: 20 mm, 23 mm, 26 mm, and 29 mm. The Evolut PRO System valve consists of an outer tissue wrap to enhance the seal the valve creates around the aortic annulus [25]. The Evolut PRO valves come in four sizes: 23 mm, 26 mm, 29 mm, and 34 mm [25]. The SAPIEN 3 valve has been associated lower risk of 30-day mortality and permanent pacemaker implantation, but the SAPIEN 3 and Evolut PRO have been shown to be comparable for risk of stroke, major vascular complications, device success, and moderate–severe residual aortic regurgitation [26]. Other valves include the ACURATE neo, the Portico, and the Myval. The ACURATE neo is a self-expanding valve and was assessed in the SCOPE 1 and 2 trials to investigate non-inferiority to the SAPIEN 3 and CoreValue Evolut valves, respectively [27,28]. It did not meet non-inferiority compared to the SAPIEN 3 nor the CoreValue Evolut valves in regards to the study endpoints of overall safety and clinical outcomes [27,28]. The Portico valve is another self-expanding valve and its efficacy was assessed within the PORTICO IDE trial [29]. The PORTICO IDE trial is ongoing, but published results compared this valve to commercially available valves within the SAPIEN and Evolut series. These results showed that the Portico valve met non-inferiority for reaching the primary safety endpoint within 30 days and the primary efficacy endpoint at 1 year. However, the Portico valve did not meet superiority criteria for any endpoint when compared to commercially available valves [29]. Finally, the Myval valve is a balloon-expandable valve that was compared to the SAPIEN and Evolut series valves in the LANDMARK non-inferiority trial [30]. This trial discovered that the Myval valve met non-inferiority criteria in its primary endpoint of safety and effectiveness at 30 days compared to contemporary valves [30].

### 4.2. Ross Procedure

The Ross procedure, which has fluctuated in and out of favor ever since it was first introduced in the 1960s, is a notable option for AVR. The procedure consists of the removal of the diseased aortic valve, followed by the excision of the patient’s own pulmonic valve and transplanting it into the aorta. The pulmonic valve is then replaced with a homograft [6]. An autograft provides numerous benefits in both short-term and particularly long-term outcomes as opposed to mechanical and bioprosthetic AVR. In a network meta-analysis conducted by Yokoyama et al., patients who underwent the Ross procedure had lower rates of pacemaker placement within 30 days of the procedure when compared to mechanical AVR (M-AVR) patients [31]. Long-term outcomes as measured through the studies (~7.4 years) found that those who underwent the Ross procedure had significantly lower all-cause mortality and long-term strokes when compared to both bioprosthetic AVR (B-AVR) and M-AVR [31]. Additionally, the Ross procedure resulted in significantly lower rates of reintervention and endocarditis when compared to B-AVR, and major bleeding when compared to M-AVR [31]. Patients who received the Ross procedure do not require long-term anticoagulation therapy and maintain neurohumoral responsiveness, indicating a more promising quality of life [31]. These outcomes are of particular interest in the young adult and child population as the Ross procedure has been linked to greater survival, decreased risk of complications, growth potential, and avoidance of anticoagulation therapy [32]. The Ross procedure is currently the mainstay recommendation for aortic valve replacement in adolescents and young adults [6]. Limitations of the Ross procedure include higher rates of reintervention and increased development of aortic regurgitation, especially for those with a larger aortic annulus or a mismatch between the aortic and pulmonic roots [31]. Additionally, the surgery has numerous absolute and relative contraindications, including Marfan syndrome, pulmonary valve disease, immune disorders, 3-vessel coronary artery disease (CAD), rheumatic valve disease, and a dilated aortic root [33]. Despite these limitations, the Ross procedure is a highly promising alternative option for select patient populations and thus requires advanced patient education and thorough discussion.

## 5. Specific Clinical Scenarios

### 5.1. Annular Size

Annular sizing plays a crucial role in achieving successful outcomes in AVR. Selecting the correct valve size is essential, as a patient’s unique annular dimensions often determine which procedure, SAVR or TAVR, will be most beneficial [34,35].

For patients with a smaller annulus, issues with patient–prosthesis mismatch (PPM) are common. This mismatch occurs when the valve is too small relative to body size, leading to elevated gradients across the valve and potentially reducing survival rates [36]. When the patient is undergoing SAVR, an annular enlargement procedure can be performed (such as Manougian, Nicks, Konno, or Y technique) in order to place a larger valve [37]. In TAVR specifically, accurate annular measurements are critical to avoiding complications such as paravalvular leaks, valve migration, or annular rupture [38]. Despite this, post-operative complications arise in patients with a small annulus who undergo TAVR or SAVR.

Patients with larger annuli, on the other hand, may face challenges with device positioning and anchoring [39]. Patients with larger annuli who undergo TAVR are at higher risk for paravalvular regurgitation, which has been linked to worse long-term outcomes if left untreated [40]. Therefore, these patients may be better suited for SAVR [41].

Regardless of annulus size, when the prosthesis and annulus are misaligned, structural valve deterioration may occur over time, negatively impacting patient outcomes and potentially requiring additional interventions [42,43]. Proper annular sizing in both TAVR and SAVR is associated with improved hemodynamics, reduced reintervention rates, and a better quality of life post-replacement.

### 5.2. Bicuspid Aortic Valve

A bicuspid aortic valve (BAV) is the most common congenital heart defect in adults, affecting 1–2% of the population [44,45,46]. There are many etiologies leading to a BAV, but the most common is when two of the three aortic valve leaflets fuse; specifically the left and right cusps [44]. It is recommended patients are evaluated in specialized BAV clinics due to the increased complexity of their care [44]. Kang et al. found that depending on which leaflets are fused, the degree of valvular dysfunction and aortic enlargement differed [45]. Importantly, more than half of patients with BAV will receive AVR in their lifetime [47]. Furthermore, bicuspid aortopathy, considered the outcome of aortic dilatation and aneurysm in patients with BAV, is seen in up to 50% of patients, and about 1% can have an aortic dissection [44]. As patients with BAV are also at a higher risk of AS, AI, and endocarditis, it is highly important to follow their disease progression and ensure interventions are happening at ideal times [44,46].

Determining the optimal time for surgical intervention is still a debate for this patient population. For example, deciding to wait to intervene until symptoms begin is one question that still needs more research [47]. SAVR is the recommended procedure for this patient population as the anatomy of the valve can be difficult for TAVR, but as sutureless valves continue to improve, this may become another option [47]. More research is needed to determine when it is best to surgically intervene in this patient population.

### 5.3. Concomitant Procedures

Current guidelines for determining whether a patient can undergo concomitant procedures during AVR emphasize a comprehensive, individualized risk assessment. This assessment considers factors such as age, frailty, comorbidities, and input from a multidisciplinary heart team to guide the decision-making process, ensuring that the addition of procedures to AVR will provide a clear benefit to the patient. The ACC/AHA and the ESC/EACTS each provide key recommendations in this regard.

For patients with significant coronary artery disease (CAD)—typically defined as >50% stenosis in major coronary arteries—the guidelines recommend combining coronary artery bypass grafting (CABG) with SAVR, as this approach has been shown to improve long-term survival [6,48,49]. In cases where patients have an ascending aortic diameter of ≥4.5 cm, guidelines also advocate for simultaneous aortic aneurysm repair to reduce the risk of rupture, including in patients with BAV [49,50,51,52,53,54,55,56,57,58]. Patients with severe asymptomatic AS who are receiving cardiac surgery for other reasons are also recommended to receive AVR [6]. Studies suggest that for patients undergoing SAVR for AS who also have severe primary mitral regurgitation, mitral valve repair is advised at the time of surgery [49]. Lastly, for patients undergoing surgery for CABG, the ascending aorta, or another valve, studies suggest concomitant AVR should be performed for both symptomatic and asymptomatic aortic regurgitation to optimize outcomes and reduce the need for future interventions [49,59,60].

### 5.4. Lifelong Management of Valve Disease

After TAVR, managing conduction abnormalities is crucial due to the risk of complications. Patients with preexisting conduction abnormalities, such as right bundle branch block (RBBB) or significant aortic valve calcification, are at higher risk. They benefit from 48 to 72 h of ECG monitoring post-procedure, and temporary pacing may be used during this period to address transient blocks [61,62]. If persistent high-grade AV block or symptomatic bradycardia develops, permanent pacemaker implantation is considered based on the likelihood of conduction recovery [61]. For mild, potentially reversible blocks, a conservative approach with extended monitoring helps avoid unnecessary permanent pacing [61,62]. Regular follow-up and ECGs are essential to monitor for late-onset conduction issues and confirm stability.

After AVR, the choice of antithrombotic and anticoagulant therapy depends largely on the type of valve and individual patient risk factors. Mechanical valves require VKA with a target INR of 2.5 to 3.5 depending on the valve position and patient-specific factors. Aspirin may be added in high-risk cases, though bleeding risks must be carefully managed [63]. In contrast, bioprosthetic valves require less intensive anticoagulation. Typically, low-dose aspirin alone is recommended long-term [63]. Dual antiplatelet therapy (DAPT) with aspirin and clopidogrel can be be used temporarily after TAVR to lower thromboembolic risk [63]. Patient-specific factors, such as a history of atrial fibrillation or thromboembolism, are critical in guiding long-term anticoagulation needs, as these patients may require ongoing anticoagulation regardless of valve type. Clinical follow-ups to assess bleeding risks are essential to ensure that therapy remains safe and effective. This individualized approach balances the need for thrombotic prevention with the risk of bleeding complications.

## 6. Outcomes Between Surgical Aortic Valve Replacement and Transcatheter Aortic Valve Replacement

### 6.1. Long-Term Durability

The debate surrounding the long-term outcomes of valve replacement is normally regarding bioprosthetic valves, as mechanical valves are known for their longevity [14]. As TAVR extends to low-risk patients with longer survival as a surgical option, many studies are investigating its durability. In 2015, a simulation study was performed to assess the durability of TAVR valves to SAVR valves. This study reported that the durability of TAVR valves may be significantly reduced compared to SAVR. Using the knowledge that SAVR valves have a durability of approximately 20 years, they estimated the durability of TAVR valves to be up to 7.8 years [64]. Aldalati et al. compared SVD between SAVR and TAVR. They found that there was no difference in SVD rates nor hemodynamic changes between SAVR and TAVR up to 8 years post-procedure [65]. Blackman et al. also found similar encouraging results in their study investigating the long-term durability of TAVR. They reported that 91% of their patients did not have SVD in a median follow-up time of 5.8 years [66]. Long-term outcomes investigating the durability of TAVR valves are still lacking. While short and intermediate-term outcomes are promising, without the knowledge of how long these valves last, it is difficult to promote TAVR intervention in younger, low-risk patients.

### 6.2. Stroke

Multiple meta-analyses have demonstrated no difference in risk of stroke between TAVR and SAVR across many patient characteristics [67,68,69,70]. TAVR is associated with a lower risk of stroke for patients who have had prior cardiac surgery [71,72]. For high-risk surgical patients, TAVR has been associated with a greater risk of stroke up to three years post-intervention [73]. The PARTNER 3 (Safety and Effectiveness of the SAPIEN 3 Transcatheter Heart Valve in Low Risk Patients with Aortic Stenosis) Trial showed a lower risk of stroke in TAVR vs. SAVR patients at 1-year follow-up, but not at 2 years [74].

### 6.3. Length of Stay

TAVR’s shorter length of inpatient stay in comparison to SAVR makes TAVR a favorable option [75]. The average length of stay in TAVR patients is about 6.2 days vs. 10.2 days for SAVR, though this is an older study [76]. A meta-analysis in 2017 examining five randomized controlled trials reported the average length of inpatient stay to be 9.6 and 12.2 days for TAVR and SAVR, respectively [77]. For female patients, the average length of stay following TAVR has been reported as 7.8 days, compared to 10.5 days for SAVR [78]. When TAVR is performed concurrently with PCI, on average, it has a shorter length of stay than SAVR with concurrent CABG [79]. Furthermore, the length of stay for TAVR has decreased over time [80]. In 2013, the length of stay for TAVR and SAVR were comparable; however, by 2016, the length of stay for TAVR was significantly shorter [80]. The length of stay for TAVR has continued to decrease since the studies above were published. Recent evidence from the Cleveland Clinic revealed that 85.9% of patients who underwent transfemoral TAVR in 2020 were discharged the same day or the following day [81].

### 6.4. Conduction Abnormalities

Following intervention, conduction abnormalities are more common in patients who underwent TAVR than SAVR [74]. A conduction abnormality is a common indicator for pacemaker placement following TAVR [82]. As TAVR carries a higher risk for conduction abnormalities than SAVR, TAVR is also associated with elevated 30-day risk for pacemaker implantation [83]. The risk of pacemaker implantation for TAVR patients remains elevated when compared to minimally invasive SAVR [84]. When examining 2-year outcomes following the NOTION trial, 41.3% of patients randomized to TAVR underwent pacemaker implantation compared to 4.2% in the SAVR group [85].

### 6.5. Myocardial Infarction

At 30 days post-intervention, the risk of myocardial infarction (MI) following TAVR is less than half of the risk of MI following SAVR; however, the risk of MI is comparable in both, at 1 and 2-year follow-up [83,85]. These results are inconsistent with a multicenter randomized trial that demonstrated no significant difference in the risk of MI between SAVR and TAVR at 1 month, 1 year, and 2-year follow-up [42]. It has been shown that females and patients with diabetes who undergo TAVR vs. SAVR are less likely to experience an MI [78,86].

### 6.6. Death

TAVR and SAVR at 1 month and 1 year have been demonstrated to have comparable mortality rates [83]. This comparable risk of mortality extends to 2 years, as demonstrated by the NOTION trial studying TAVR vs. SAVR in patients with severe AS [85]. A recent meta-analysis investigating low-risk patients with severe AS revealed all-cause mortality at 10-year follow-up to be 45.8% and 56.6% for TAVR and SAVR patients, respectively. [67]. The risk for cardiovascular death following TAVR or SAVR does not differ significantly at 1- and 5-year follow-up [87].

### 6.7. Need for Repeat Intervention

There is evidence to show that TAVR carries a larger risk for repeat intervention than SAVR [88]. The PARTNER 2 study in 2016 showed an increased risk for reintervention following TAVR compared to SAVR [42]. More recent studies have shown a similar increased risk for reintervention following TAVR compared to SAVR, but these were not significant findings [89,90]. Recent evidence shows that the increased risk for reintervention following TAVR is concentrated within the first year post-procedure [91]. The need for repeat intervention following TAVR is decreasing over time, with patients in the early 2010s having a 50% increased risk of reintervention compared to patients treated more recently [92]. Suspected reasons for reintervention in TAVR include paravalvular leak, valve embolization, and coronary obstruction [92]. Reintervention with SAVR following TAVR carries an increased risk of mortality compared to repeat SAVR after prior SAVR [93].

### 6.8. Prosthetic Valve Endocarditis

Prosthetic valve endocarditis (PVE) is a rare yet fatal consequence following both SAVR and TAVR. The rate of PVE post-SAVR ranges from 0.3% to 1.2%, while post-TAVR, it ranges from 0.6% to 3.4% [94]. For SAVR, endocarditis is a common cause for reintervention [91]. Of note, bioprosthetic valves are more commonly associated with PVE, which is likely due to bacterial invasion of degenerated valve leaflets [94]. TAVR is associated with early PVE, defined as occurring in less than 2 months, and higher readmission rates at 30 and 90 days. However, there is no significant difference in PVE rates between SAVR and TAVR [94].

Imaging modalities to identify PVE is more difficult in TAVR than SAVR. This is likely due to the stented frame causing shadowing on echocardiography, as well as the native valve still being present [94]. Mangner et al. stated that negative echocardiography may be present in up to 15% of patients with PVE post-TAVR [95]. Therefore, beginning antibiotic treatment with a high suspicion of PVE, even with negative imaging, is reasonable in post-TAVR patients [94,95].

## 7. Importance of the Multidisciplinary Heart Valve Team

The 2020 VHD guidelines list the multidisciplinary Heart Valve Team (MDT) and Heart Valve Centers as having high importance regarding treatment decisions in VHD. Patients with severe VHD are more strongly recommended to use this service, but asymptomatic patients, patients with multiple comorbidities, or any patient who is deciding on which treatment option is best for them, are also recommended to consult an MDT. This team consists of many members, including a primary care cardiologist, VHD cardiac specialists, specialists in cardiac imaging, interventional cardiologists, and more. The goal of this team is to thoroughly discuss treatment options with patients and allow for shared decision-making so the best outcome is obtained [6].

## 8. Discussion

Many patients with aortic valve disease will require surgical intervention to improve hemodynamics. The primary intervention options include SAVR and TAVR, which are comparable in their outcomes. Valve-in-valve (VIV) techniques are increasing in popularity as well. This procedure allows for a transcatheter rather than a surgical approach when the bioprosthetic valve has degenerated and reintervention needs to occur [96]. The main adverse events include malpositioning of the valve, coronary obstruction, and elevated post-procedure gradient [96,97]. The largest considerations for patients receiving this procedure are the internal diameter of the current valve, the etiology of SVD, and the type of valve that needs to be implanted. Studies have found an association between small internal diameter sizes, <21 mm, and worse survival for patients [96,97]. VIV is a valuable option for those who need reintervention and fit the surgical considerations for positive outcomes.

The ability to adhere to VKA therapy and age are the largest deciding factors for valve selection. Mechanical valves have increased longevity; however, the patient will need to be on chronic VKA therapy, placing them at a higher bleed risk. There is a current trend towards using more bioprosthetic valves, possibly due to the increasing age of those receiving AVR, patients not wanting to be on chronic VKA, and bioprosthetics having better hemodynamic stability [17]. The age cutoffs listed in the ACC/AHA guidelines are reasonable with current research, especially with current knowledge surrounding the longevity of bioprosthetic valves. However, it is important to note that shared decision-making is a dominant factor. As VIV surgery becomes more common, those with failing bioprosthetic valves have a noninvasive surgical option for reoperation. Either valve is reasonable to use with any age, once all the considerations are discussed with the patient, including the likelihood of reoperation.

## Figures and Tables

**Table 1:** Adaptation of the 2020 ACC/AHA Guidelines for Class of Recommendation and Level of Evidence.

Class of Recommendation (COR)	Level of Evidence (LOE)
Class 1 (Strong): Benefit >>> Risk	Level A: high quality of evidence from multiple randomized control trials
Class 2a (Moderate): Benefit >> Risk	Level B: moderate quality of evidence from multiple randomized control trials or nonrandomized studies
Class 2b (Weak): Benefit ≥ Risk	Level C: limited data studies containing limitations of design or execution OR consensus of expert opinion based on clinical experience
Class 3 with LOE A or B (No benefit, Moderate): Benefit = Risk	
Class 3 without LOE A or B (Harm, Strong): Risk > Benefit	

**Table 2:** Adaptation of the 2020 ACC/AHA Guidelines for Patients with Aortic Stenosis.

Class of Recommendation	Level of Evidence	Recommendations
1	A	Anyone with signs or symptoms of AS is recommended to be evaluated with TTE, including assessment of disease progression, to accurately determine prognosis and evaluate the need for valve intervention
1	A	Adults with severe symptomatic AS are recommended to receive AVR
1	A	Any patient receiving a bioprosthetic AVR who is <65 years old or has a >20-year life expectancy is recommended to receive SAVR
1	A	Patients receiving a bioprosthetic AVR aged 65 to 80 years old with no contraindication to either SAVR or TAVR are recommended to use shared decision-making
1	A	Any patient receiving a bioprosthetic AVR who is >80 years old or has a life expectancy of <10 years and no anatomic contraindications is recommended to receive transfemoral TAVR
1	B	Adults with severe asymptomatic AS with LVEF < 50% are recommended to receive AVR
1	C	In patients receiving AVR, valve type should be a shared decision-making process
1	C	Any patient receiving AVR with a contraindication to VKA therapy is recommended to receive a bioprosthetic valve
2b	B	Patients < 50 years old who have appropriate anatomy and prefer a bioprosthetic valve may receive a pulmonic autograft for their AVR (Ross procedure) at a Comprehensive Valve Center

Acronyms: ACC: American College of Cardiology, AHA: American Heart Association, AS: Aortic Stenosis, TTE: Transthoracic Echocardiogram, AVR: Aortic Valve Replacement, LVEF: Left Ventricular Ejection Fraction, VKA: Vitamin K Antagonist, SAVR: Surgical Aortic Valve Replacement, TAVR: Transcatheter Aortic Valve Replacement.

**Table 3:** Adaptation of the 2020 ACC/AHA Guidelines for Patients with Aortic Insufficiency.

Class of Recommendation	Level of Evidence	Recommendations
1	B	Anyone with signs or symptoms of AI is recommended to be evaluated with TTE, including assessment of disease progression, to accurately determine prognosis and evaluate the need for valve intervention
1	B	In patients with severe symptomatic AI, AVR is recommended irrespective of LV systolic function
1	B	In patients with severe asymptomatic chronic AI and LVEF < 55%, AVR is recommended
1	C	Patients with severe AI who are receiving cardiac surgery for other reasons are recommended to receive concomitant AVR

Acronyms: ACC: American College of Cardiology, AHA: American Heart Association, AI: Aortic Insufficiency, TTE: Transthoracic Echocardiogram, AVR: Aortic Valve Replacement, LV: Left Ventricle, LVEF: Left Ventricular Ejection Fraction.

**Table 4:** Adaptation of the 2020 ACC/AHA Guidelines for Patients with Infective Endocarditis.

Class of Recommendation	Level of Evidence	Recommendations
1	B	A Heart Valve Team should assist in the decisions surrounding surgical intervention for IE
1	B	Early surgery prior to completion of antibiotics is recommended for those with complicating valvular dysfunction
1	B	Early surgery prior to completion of antibiotics is recommended for those with complications of conduction abnormalities, annular or aortic abscess, or destructive lesions
1	B	Early surgery is recommended for those with persistent infection (>5 days after beginning antibiotic treatment)

Acronyms: ACC: American College of Cardiology, AHA: American Heart Association, IE: Infective Endocarditis.

**Table 5:** Adaptation of the 2020 ACC/AHA Guidelines for Valve Selection.

Class of Recommendation	Level of Evidence	Recommendations
1	C	Shared decision-making should be used in any patient requiring valve replacement
1	C	Any patient who has a contraindication to VKA is recommended to receive a bioprosthetic valve
2a	B	Patients < 50 years old without a contraindication to VKA are recommended to receive a mechanical valve
2a	B	Patients between 50 and 65 years old without a contraindication to VKA can receive either a mechanical or bioprosthetic valve
2a	B	Patients > 65 years old are recommended to receive a bioprosthetic valve

Acronyms: ACC: American College of Cardiology, AHA: American Heart Association, VKA: Vitamin K Antagonist.

**Table 6:** Adaptation of the 2020 ACC/AHA Guidelines for Patients with Bicuspid Aortic Valve.

Class of Recommendation	Level of Evidence	Recommendations
1	B	Anyone with BAV is recommended to be evaluated with TTE to assess morphology and disease status to evaluate the need for valve intervention
2b	B	Patients with BAV and severe symptomatic AS may receive AVR at a Comprehensive Valve Center using TAVR over SAVR
2b	C	Patients with BAV and severe AI may receive AVR if the surgery is performed at a Comprehensive Valve Center

Acronyms: ACC: American College of Cardiology, AHA: American Heart Association, BAV: Bicuspid Aortic Valve, TTE: Transthoracic Echocardiogram, AS: Aortic Stenosis, AVR: Aortic Valve Replacement, TAVR: Transcatheter Aortic Valve Replacement, SAVR: Surgical Aortic Valve Replacement, AI: Aortic Insufficiency.

**Table 7:** Adaptation of the 2020 ACC/AHA Guidelines for Concomitant Procedures.

Class of Recommendation	Level of Evidence	Recommendations
1	B	Patients with severe asymptomatic AS who are receiving cardiac surgery for other reasons are recommended to receive concomitant AVR
2a	C	Patients with significant AS and complex or multivessel CAD are recommended to receive SAVR and CABG concomitantly

Acronyms: ACC: American College of Cardiology, AHA: American Heart Association, AS: Aortic Stenosis, AVR: Aortic Valve Replacement, CAD: Coronary Artery Disease, SAVR: Surgical Aortic Valve Replacement, CABG: Coronary Artery Bypass Graft.

**Table 8:** Adaptation of the 2020 ESC/EACTS Guidelines for Concomitant Procedures.

Class of Recommendation	Level of Evidence	Recommendations
1	C	Patients receiving CABG, surgery on the ascending aorta, or surgery of another valve, are recommended to receive SAVR if severe AS is present
2a	C	Patients receiving AVR are recommended to receive concomitant aortic root/ascending aorta replacement if the diameter is ≥4.5 cm

Acronyms: ESC: European Society of Cardiology, EACTS: European Association for Cardio-Thoracic Surgery, CABG: Coronary Artery Bypass Graft, SAVR: Surgical Aortic Valve Replacement, AS: Aortic Stenosis, AVR: Aortic Valve Replacement.

**Table 9:** Adaptation of the 2020 ACC/AHA Guidelines for the Multidisciplinary Heart Valve Team and Heart Valve Centers.

Class of Recommendation	Level of Evidence	Recommendations
1	C	Evaluation by an MDT should be completed for those with severe VHD when considering intervention

Acronyms: ACC: American College of Cardiology, AHA: American Heart Association, MDT: Multidisciplinary Heart Valve Team, VHD: Valvular Heart Disease.

## Data Availability

The original contributions presented in this study are included in the article; further inquiries can be directed to the corresponding author.
